# Adrenal Function Testing Following Hormone Therapy for Infantile Spasms: Case Series and Review of Literature

**DOI:** 10.3389/fneur.2015.00259

**Published:** 2015-12-08

**Authors:** John R. Mytinger, Sasigarn A. Bowden

**Affiliations:** ^1^Department of Pediatrics, Division of Pediatric Neurology, Nationwide Children’s Hospital, The Ohio State University, Columbus, OH, USA; ^2^Department of Pediatrics, Division of Pediatric Endocrinology, Nationwide Children’s Hospital, The Ohio State University, Columbus, OH, USA

**Keywords:** infantile spasms, West syndrome, adrenal insufficiency, adrenal function, adrenal stimulation testing, cortisol, adrenocorticotropic hormone, prednisolone

## Abstract

Prednisolone and adrenocorticotropic hormone (ACTH) are “hormone” therapies for infantile spasms. There is limited data on the occurrence of decreased adrenal reserve or signs of clinical adrenal insufficiency after hormone therapy. This is a retrospective medical record review of patients referred to our Infantile Spasms Program. Our standardized infantile spasms management guideline began in September 2012 and initially included a post-hormone laboratory assessment of adrenal function. Medical records were assessed for hormone treatments, adrenal function testing, and signs of adrenal insufficiency. Forty-two patients who received one or both hormone therapies met inclusion criteria. A post-hormone laboratory assessment of adrenal function was done in 14 patients. Of these 14 patients, 2 had an abnormal laboratory assessment of adrenal function, both by adrenal stimulation testing – one after ACTH and one after prednisolone. One patient received hydrocortisone replacement and the other received stress dose hydrocortisone as needed; neither patient developed signs of adrenal insufficiency. Another patient treated with both types of hormone therapy in tandem, who did not have a post-hormone laboratory assessment, developed signs of mild adrenal insufficiency and required replacement hydrocortisone. Our study suggests that adrenal suppression can occur after modern hormone therapy regimens. We found two patients with abnormal adrenal function testing after hormone therapy and another patient with signs adrenal insufficiency. Given the seriousness of adrenal crisis, caregiver education on the signs of adrenal insufficiency is critical. Greater vigilance may be indicated in patients receiving both types of hormone therapy in tandem. Although a routine post-hormone laboratory assessment of adrenal function may not be feasible in all patients, replacement or stress dose hydrocortisone is necessary for all patients with suspected adrenal insufficiency.

## Introduction

Infantile spasms are seizures that most often begin in the first year of life. Early and successful treatment of infantile spasms and the associated epileptic encephalopathy can improve developmental and seizure outcomes (1, 2). Adrenocorticotropic hormone (ACTH) and oral corticosteroids (most commonly prednisolone) are types of “hormone” therapies that are commonly used first-line treatments for patients with infantile spasms (3). Some infantile spasms patients who do not respond to ACTH will respond to oral corticosteroids, and vice versa (4). Thus, some clinicians switch to the alternative hormone therapy after a patient fails to respond to the first hormone therapy. In the United States, the duration of hormone therapy with ACTH or prednisolone is about 1 month (3, 5–7). Dosing regimens vary, but clinicians in the United States often use “high-dose” hormone therapy (3). High-dose regimens typically begin with 150 IU/m^2^/day for natural gel ACTH (5) and 40–60 mg/day for oral prednisolone (8).

The risk of secondary adrenal insufficiency due to the suppression of the hypothalamic–pituitary–adrenal (HPA) axis following long-duration high-dose corticosteroids is well known (9). Suppression of the HPA axis may be partial or total, as a result of adrenal gland atrophy, with great individual variability (10). Even short-duration corticosteroid therapy (e.g., 10 days) can lead to the suppression of the HPA axis (11). When steroid therapy is withdrawn in patients with suppressed adrenal function, acute adrenal insufficiency or Addisonian crisis may occur, which is a serious and potentially life-threatening condition. Signs of mild adrenal insufficiency are non-specific, but can include nausea, tiredness, poor feeding, and lethargy in young children. Signs of severe acute adrenal insufficiency can include vomiting, diarrhea, fever or hypothermia, hypotension, hypoglycemia, shock, and coma. No study has assessed adrenal function or the incidence of clinical adrenal insufficiency following the treatment of infantile spasms with modern regimens of high-dose corticosteroid therapy.

Impaired pituitary ACTH response has been shown in adults following exogenous ACTH administration (12). Exogenous ACTH stimulates the adrenal gland, resulting in hypercortisolism, as evidenced in ACTH-treated infants who develop Cushingoid appearance as a result of this effect. The mechanism of decreased pituitary reserve following exogenous ACTH therapy has been postulated to result from hypercortisolemia, which suppresses pituitary ACTH secretion; this causes rapid involution of the adrenal cortex after stopping ACTH treatment and reduced responsiveness to ACTH stimulation (13).

There is very limited data available on the risk of suppression of the HPA axis following modern regimens of ACTH therapy for infantile spasms. There have been three small infantile spasms studies totaling 24 patients treated with ACTH who had a post-treatment assessment of adrenal function (13–15). Several patients were reported to have decreased pituitary and/or adrenal reserve after stopping ACTH therapy (13–15). In these three studies, adrenal function was assessed either soon after stopping ACTH or up to 2 weeks later; these studies did not assess the occurrence of clinical adrenal insufficiency in the months following treatment. Although two large series of infantile spasms patients treated with synthetic or natural ACTH did not report any cases of clinical adrenal insufficiency following treatment with ACTH, these studies did not assess adrenal function (16, 17). It is important to note that symptoms of clinical adrenal insufficiency in patients with a suppressed HPA axis may not be clinically evident without stress (e.g., febrile illness). It is also unknown if the risk of adrenal insufficiency is increased, if the alternative hormone therapy is used after the first hormone therapy, either when one is used immediately after the other (i.e., in tandem) or following a brief hiatus between hormone therapies.

At Nationwide Children’s Hospital, we created and implemented a standardized management guideline for infantile spasms in September 2012. Given the uncertainty of adrenal insufficiency following hormone therapy for infantile spasms, and a personal experience of clinical adrenal insufficiency following corticosteroid therapy in a patient with infantile spasms (18), our guideline initially included a post-hormone endocrinologist-guided laboratory evaluation of adrenal function. Here, we report our experience with monitoring of adrenal function following hormone therapy for infantile spasms as well as the occurrence of clinical adrenal insufficiency.

## Materials and Methods

### Subjects and Treatment Protocol

We carried out a retrospective medical record review of patients referred to the Infantile Spasms Program at Nationwide Children’s Hospital from September 2012 to July 2015. The medical records of patients were assessed for hormone treatments, adrenal function testing, and clinical signs of post-treatment adrenal insufficiency. The goal of this study was to determine the frequency of decreased adrenal reserve or signs of clinical adrenal insufficiency in infantile spasms patients treated with hormone therapy.

Our management guideline emphasizes the initial treatment with first-line therapies which at our program include ACTH, oral prednisolone, and vigabatrin. After physician-guided counseling, caregivers and clinicians work together to choose the desired treatment. Given that some patients who fail to respond to ACTH will respond to oral corticosteroids, and vice versa (4), some patients in this study received the alternative hormone therapy immediately after the first hormone therapy was deemed ineffective. Some patients received vigabatrin in between hormone therapies depending on the preferences for treatment. During the study period, natural gel ACTH was given at a dose of 75 IU/m^2^ twice daily for 2 weeks followed by a taper over 15 days [30 IU/m^2^/day for 3 days, 15 IU/m^2^/day for 3 days, 10 IU/m^2^/day for 3 days, and then 10 IU/m^2^/day every other day for three doses (5)] and prednisolone was given at a dose of 40–60 mg/day in three to four divided doses for 2 weeks followed by a 15-day taper [10 mg three times daily (or 10 mg four times daily for those that required a dose increase to 60 mg/day) for 5 days, 10 mg twice daily for 5 days, and then 10 mg daily for 5 days (8)].

### Assessment of the Hypothalamic–Pituitary–Adrenal Axis

From September 2012 to July 2014, our infantile spasms management guideline included a recommendation for an endocrinologist-guided laboratory assessment of adrenal function after completion of hormone therapy. Given the relatively low yield of this testing and the lack of clinical signs of adrenal insufficiency with laboratory testing in 14 patients, we subsequently discontinued these evaluations and instead relied upon clinical signs of adrenal insufficiency.

A laboratory assessment of adrenal function included an a.m. cortisol level and/or an assessment of adrenal function by glucagon stimulation test or ACTH stimulation test. Normal 8 a.m. cortisol is 3–21 μg/dl. The ACTH stimulation test was chosen more frequently than the glucagon stimulation test given that the former is of shorter duration. For both tests, a fasting state is not required. Baseline serum cortisol levels (as well as serum ACTH levels for most patients) were drawn upon placement of a peripheral indwelling intravenous line, which were then maintained by means of a heparin-lock needle. For glucagon stimulation testing, glucagon (Glucagen-Bedford Laboratories) at 0.03 mg/kg (maximum 1 mg) was given subcutaneously. Blood samples for serum glucose and cortisol were obtained at 60, 90, 120, and 150 min after glucagon administration. For ACTH stimulation testing, 1 μg of cosyntropin (ACTH-[1-24], Cortrosyn-Amphastar Pharmaceuticals) was administered intravenously. Blood samples were obtained at 20 and 40 min for the measurement of serum cortisol. Serum cortisol was measured using chemiluminescent enzyme immunoassay by our institution laboratory. Normal peak cortisol levels after stimulation testing are >18 μg/dl. Serum ACTH was measured using a chemiluminescent immunoassay, and normal levels are 6–48 pg/ml (Esoterix, Calabasas Hill, CA, USA).

### Statistics

We use descriptive statistics as well as median, mean, and range where appropriate. For patient demographics, all ages were corrected for gestational age <40 weeks.

### Ethics

This study was approved by the Nationwide Children’s Hospital Institutional Review Board. Given the study design (a retrospective medical record review), a waiver of written or verbal consent was granted by the Institutional Review Board. The authors have no conflicts of interest related to this research.

## Results

During the study period, 59 patients with infantile spasms were referred to our Infantile Spasms Program. Of these, 45 patients received one or both hormone treatments. Two patients were excluded from the analysis – one due to congenital panhypopituitarism (including HPA insufficiency) and another because he received an alternative ACTH regimen at an outside institution. The 14 patients who did not receive hormone therapy were treated initially with vigabatrin and never received hormone therapy either due to remission with vigabatrin, a contraindication to hormone therapy, or death due to the underlying etiology of infantile spasms.

The median age of infantile spasms onset for those patients receiving hormone therapy was 6 months (mean 6, range 2–18). The median age of starting hormone therapy was 7 months (mean 9, range 2–27). Eighteen of 42 (43%) received ACTH, 14/42 (33%) received prednisolone, and 10/42 (24%) patients received ACTH and prednisolone at some point during his/her treatment course (five received ACTH first and five received prednisolone first). Three patients receiving prednisolone had his/her dose increased from 40 to 60 mg/day during the course of treatment. In 5 of the 10 patients receiving both ACTH and prednisolone, the alternative hormone treatment was given after a hiatus between hormone therapies. In the other five patients, the alternative hormone treatment was given immediately after the first hormone therapy; in four of these five cases, the initial hormone therapy was stopped early (due to ongoing infantile spasms), resulting in fewer total hormone treatment days (42, 43, 49, and 50 days, respectively).

Fourteen of 42 patients (33%) had a laboratory assessment of adrenal function following hormone treatment during the period in which this practice was part of our standardized management guideline. Eleven of 14 patients had adrenal stimulation testing: 2 had glucagon stimulation testing and 9 had ACTH stimulation testing (Table 1). Patient 8 had adrenal stimulation testing following each hormone treatment (Table 1). The median interval between the last day of hormone therapy and the day of adrenal stimulation testing was 9 weeks (mean 14, range 2–18). In 11 patients undergoing adrenal stimulation testing, comprising 12 adrenal stimulation tests, 2/11 (18%) patients (Patient 6 and Patient 8) had suboptimal peak cortisol levels (Table 1). Detailed clinical data on these two patients are as follows.

**Table 1:** **Clinical and laboratory data of 11 patients who underwent adrenal stimulation testing**.

Patient number	Etiology	Corrected age at treatment initiation (months)	Hormone treatment^d^	Type of adrenal stimulation testing	Interval: last hormone dose to test (weeks)	Baseline ACTH level (NL: 6–48 pg/ml)	Baseline cortisol level^f^ (time of day)	Peak cortisol (NL >18 μg/dl)
1	HIE	10	ACTH	Glucagon	2	24	9.3 (1425)	19.9
2	PVL	6	ACTH	ACTH	18	ND	10.4 (1405)	26.5
3	TBI	6	ACTH	ACTH	2	27	8.6 (1440)	24.1
4	Unknown^b^	6	ACTH	ACTH	10	ND	10.1 (0935)	33.1
5	Stroke	5	ACTH	ACTH	8	39	15.4 (1415)	30.8
6	HIE	6	ACTH	Glucagon	10	23	6.4 (1010)	11.4^g^
7	Unknown^c^	5	ACTH	ACTH	13	25	11.6 (1022)	20.3
8^a^	Trisomy 21	8	PRED^e^	ACTH	8	7.4	3.1 (0908)	15.5^g^
		12	ACTH	ACTH	9	9.2	6.4 (1350)	23
9	IVH	8	ACTH	ACTH	8	67	8.3 (1520)	24.5
10	Unknown^b^	19	PRED	ACTH	6	ND	16.3 (1257)	35.2
11	HIE	5	PRED	ACTH	15	27	20 (1000)	26

*^a^Patient received 29 days each of PRED and ACTH (58 total treatment days)*.

*^b^Normal neurological examination at infantile spasms onset*.

*^c^Abnormal neurological examination at infantile spasms onset*.

*^d^The duration of treatment was 29 days for each drug*.

*^e^A random cortisol level 3.5 weeks after stopping PRED was undetectable (<0.9 μg/dl, 1400)*.

*^f^Normal cortisol levels in children: 0800, 1–16 years old, range 3–21 μg/dl; 1600, 1/2 the 0800 value*.

*^g^Two patients met laboratory criteria for adrenal insufficiency*.

*HIE, hypoxic ischemic encephalopathy; PVL, periventricular leukomalacia; TBI, traumatic brain injury; IVH, intraventricular hemorrhage; ACTH, adrenocorticotropic hormone; PRED, prednisolone; NL, normal; ND, not done*.

Patient 6 had infantile spasms onset at 4 months of age and had a history of severe perinatal hypoxic ischemic encephalopathy. He received a full course of ACTH therapy starting at 6 months of age and had adrenal stimulation testing with glucagon 10 weeks after stopping ACTH. His baseline (obtained at 9:50 a.m.) cortisol (6.4 μg/dl) and ACTH levels (23 pg/ml) were normal. However, his peak cortisol with glucagon stimulation testing was suboptimal at 11.4 μg/dl (normal peak cortisol >18 μg/dl). He was then started on a physiological replacement hydrocortisone (10 mg/m^2^/day) and received stress dose hydrocortisone for physical stress (febrile illness, trauma, or procedures). His hydrocortisone was slowly weaned off after being on hydrocortisone therapy for about 7 months. Repeat adrenal stimulation testing with ACTH 1 month after weaning off hydrocortisone showed a normal peak cortisol level (19.5 μg/dl) indicating adequate adrenal function. This patient never displayed definitive clinical signs of adrenal insufficiency.

Patient 8 had infantile spasms onset at 6 months of age and had trisomy 21. He received a full course of prednisolone starting at 8 months of age. Three and a half weeks after stopping prednisolone he had an undetectable (<0.9 μg/dl) random cortisol level (obtained at 2 p.m.). He was not started on maintenance hydrocortisone but adrenal stimulation testing was planned. Testing could not be performed until 4 weeks later (8 weeks after stopping prednisolone). At this time, his baseline (obtained at 9 a.m.) cortisol (3.1 μg/dl) and ACTH (7.4 pg/ml) levels were normal. However, his peak cortisol level with ACTH stimulation testing was suboptimal at 15.5 μg/dl. Given his borderline peak cortisol level, he was prescribed hydrocortisone to be given only at the time of physical stress (febrile illness, trauma, or procedures). At 12 months of age, ACTH therapy was initiated for ongoing infantile spasms. Nine weeks after stopping ACTH therapy, adrenal stimulation testing with ACTH showed a normal peak cortisol level of 23 μg/dl. This patient never displayed definitive clinical signs of adrenal insufficiency.

Three patients had only random morning cortisol levels obtained following completion of hormone therapy. In two patients, the a.m. cortisol levels were 10 and 7.2 μg/dl at 10 and 16 weeks after stopping hormone therapy, respectively. For these patients, no further endocrine testing was obtained given the absence of any clinical signs of adrenal insufficiency. The third patient was hospitalized for pneumonia and had an 8 a.m. cortisol level of 4.4 μg/dl. She was given stress dose hydrocortisone at 50 mg/m^2^/day during the course of her febrile illness and was subsequently weaned off hydrocortisone within 3 weeks. Adrenal stimulation testing was planned for this patient but the family did not return for this scheduled test.

Due to the relatively low yield of a post-hormone therapy endocrinologist-guided laboratory evaluation in 14 patients, we chose to discontinue systematic laboratory monitoring and instead rely on clinical signs of adrenal insufficiency. Subsequently, one patient with an unknown etiology of infantile spasms, but abnormal development prior to infantile spasms onset, displayed clinical signs of mild adrenal insufficiency after receiving a full course of ACTH immediately followed by a full course of prednisolone (maximum dose 40 mg/day) – a total of 2 months of hormone treatment. Increasing fatigue, decreased alertness, and poor feeding started 2 weeks after stopping prednisolone. She was treated with physiological replacement doses of hydrocortisone, resulting in clinical improvement. After 10 weeks of therapy, hydrocortisone was weaned off. However, she experienced recrudescent signs of adrenal insufficiency almost immediately after stopping hydrocortisone. Hydrocortisone was restarted and was ongoing 4 months after stopping prednisolone (the time of last follow-up).

## Discussion

This cases series includes 42 patients treated with hormone therapy for infantile spasms; 14 of these patients underwent an endocrinologist-guided laboratory assessment of adrenal function after hormone therapy. Eleven of 14 patients had adrenal stimulation testing and 2/11 (18%) patients had suboptimal peak cortisol levels. We contrast our study with three prior studies that have assessed adrenal function by stimulation testing after ACTH therapy in Table 2. In a study by Ross, 1/5 (20%) patients had abnormal adrenal stimulation testing (after the insulin tolerance test) soon after stopping natural ACTH (14). Perteentupa and colleagues reported two-thirds of their patients had abnormal adrenal stimulation testing (after combined vasopressin-synthetic ACTH) up to 2 weeks after stopping synthetic ACTH (13). Rao and Willis reported 5/9 (56%) patients with abnormal adrenal stimulation testing (after metyrapone infusion) 2 days after stopping natural ACTH (15). The insulin tolerance test used by Ross, mimicking a severe stress, has been abandoned due to the risk of hypoglycemic seizures and severe hypokalemia after treatment with glucose infusion (19, 20). The metyrapone test used by Rao and Willis is an excellent test to evaluate the integrity of adrenal function, but it is rarely performed because of the difficulty in obtaining metyrapone and the risk of precipitating an adrenal crisis (20). While the timing of HPA testing in all previous studies ranged from the next day to 2 weeks after ACTH withdrawal, our study assessed adrenal function at median interval of 9 weeks. The longer post-hormone adrenal function assessment adds to the limited literature on this topic. We found two patients with suppressed adrenal function 8 and 10 weeks after stopping natural ACTH and prednisolone, respectively. The length of HPA suppression in these two patients is similar to previous study in children with leukemia, reporting that a 4-week course of glucocorticoids resulted in suppression of the HPA axis for up to 8 weeks after discontinuation (21). While both patients in our study later had normal adrenal stimulation testing, our findings highlight the importance monitoring patients for signs of adrenal insufficiency after hormone therapy.

**Table 2:** **Four infantile spasms studies that report decreased pituitary or adrenal reserve with stimulation testing after hormone therapy**.

Study	Ross (14)	Perheentupa et al. (13)	Rao and Willis (15)	Mytinger and Bowden (22)
Number of patients	5	10	9^c^	11
Age of IS onset	Unknown, but IS onset, diagnosed or treated: 8 months to 8 years	Unknown, but admitted for treatment at a mean of 9 months (range 5–22)	Unknown, but IS onset, diagnosed, or treated: median 5.5 months (mean 7, range 1.8–16)	Median 6 months (mean 8, range 5–19)
Treatment	Natural ACTH	Synthetic ACTH	Natural ACTH	Natural ACTH or prednisolone
Dose	1.1–9 IU/kg/day	80 IU (mean 9.5 IU/kg, range 6.6–13.1)	20–40 IU/day (50–160 IU/m^2^/day)^d^	150 IU/m^2^/day ACTH or 40–60 mg/day PRED
Treatment duration range (days)	30–90 (“1–3 months”)	42	30–66	29
Type of testing	Insulin infusion, then β-endorphin and serial cortisol levels	Combined vasopressin-synthetic ACTH, then serial cortisol levels	Metyrapone infusion, then cortisol level	Synthetic ACTH or glucagon infusion, then serial cortisol levels
Timing of testing from last dose of hormone	Immediately after completion of ACTH	3 days, 1, and 2 weeks after completion of ACTH	2 days after completion of ACTH	Median 9 weeks (mean 14, range 2–74) weeks after last hormone dose
↓ Hypothalamic–pituitary reserve or adrenal reserve	1^a^/5	3 days: 1^b^/6	5^e^/9	2^f^/11
		1 week: 2^b^/4		
		2 weeks: 5^b^/8		
Number with clinical AI	Not discussed	Not discussed	Not discussed	1 (mild)

*^a^Patient had normalization of hypothalamic–pituitary–adrenal axis function 6 weeks after abnormal testing*.

*^b^Testing was subnormal at 2 hour post ACTH*.

*^c^One of 10 patients in the study did not have a post-treatment HPA axis assessment*.

*^d^No taper was used*.

*^e^Only one patient had subsequent adrenal stimulation testing, which was normal about 8 weeks after the last dose of ACTH*.

*^f^Adrenal stimulation testing was normal with the next evaluation 18.5 and 9 months after the last hormone dose in these two patients*.

*IS, infantile spasms; ACTH, adrenocorticotropic hormone; IU, international units; PRED, prednisolone; ↓, decreased; AI, adrenal insufficiency*.

It is of interest that both patients with abnormal adrenal stimulation testing in our study had normal random cortisol and ACTH levels prior to abnormal adrenal stimulation testing. This suggests that random cortisol and ACTH levels are less sensitive to the detection of reduced adrenal reserve compared to adrenal stimulation testing. This can be explained by a study by Graber and colleagues who evaluated recovery of the HPA axis after withdrawal of chronic corticosteroid therapy (23). These authors demonstrated that ACTH was the first to normalize, followed by morning serum cortisol, and finally cortisol after adrenal stimulation testing with ACTH. Therefore, at the time of the adrenal stimulation testing after hormone therapy, the serum ACTH and cortisol levels may have already normalized (depending on the timing of assessment). Under normal unstressed conditions, single ACTH concentration is not useful to detect secondary adrenal suppression (24).

Given the relatively low incidence of abnormal adrenal function testing after testing 14 patients, we removed the laboratory assessment of adrenal function from our standardized infantile spasms management guideline. Subsequently, one patient developed signs of mild adrenal insufficiency. While speculative, it is possible that the tandem use of the two hormone therapies predisposed this patient to develop adrenal insufficiency. Although another patient treated with the two hormone therapies in tandem was proactively treated with stress dose hydrocortisone during a hospitalization for pneumonia, she was never formally diagnosed with adrenal insufficiency; this patient did not undergo adrenal stimulation testing, and she never required subsequent replacement or stress dose hydrocortisone. Three other patients treated with both hormone therapies in tandem, and five patients treated with both hormone therapies with a hiatus between hormone therapies did not develop clinical signs of adrenal insufficiency. Based on a single patient with clinical adrenal insufficiency, it is not possible to delineate the risk of adrenal insufficiency in patients receiving one hormone therapy after another for the treatment of infantile spasms.

The post-hormone low-dose synthetic ACTH stimulation test utilized for most patients in our study is a sensitive and convenient test for secondary adrenal insufficiency, although caution is advised for possible false negative results in patients with recent or mild secondary adrenal insufficiency, as the adrenal glands may respond normally to exogenous ACTH. Glucagon stimulation testing is a sensitive test for evaluating adrenal function and is not associated with the risks of insulin-induced hypoglycemia. Glucagon administration causes an increase in blood glucose, which then evokes an endogenous insulin response, resulting in a fall in blood glucose that stimulates a counter-regulatory hormone response that includes cortisol (25).

Limitations of our study include our retrospective study design, an overall small sample size with a limited number of patients undergoing a post-hormone laboratory assessment of adrenal function, and differing preferences among endocrinologists in the type of adrenal function testing utilized. Despite these limitations, our study adds to this limited body of literature and is the only study to assess adrenal function following consistently utilized modern hormone treatment regimens for infantile spasms. Given the potential risk of life-threatening adrenal crisis (20), we recommend that clinicians should be vigilant in monitoring for signs of adrenal insufficiency for at least 3 months (or longer) after withdrawal of hormone therapy. Ideally, dynamic adrenal function testing, with ACTH stimulation testing or glucagon stimulation testing, would be obtained in all patients. However, this may not be feasible in routine practice. Alternatively, prophylactic coverage with stress dose hydrocortisone should be given in times of physical stress (e.g., febrile illness, trauma, or procedures), as suggested by Chamberlin and colleagues (10). It is critical that caregivers should be educated on the signs of adrenal insufficiency. Further studies are needed to evaluate the effectiveness of this approach in prevention of morbidity associated with HPA suppression in infantile spasms patients treated with hormone therapy.

### Summary and Conclusion

Our study suggests that adrenal suppression can occur after treatment of modern hormone therapy regimens. Although we found relatively few patients with abnormal adrenal stimulation testing (18%) and only one additional patient with clinical signs adrenal insufficiency, given the seriousness of adrenal crisis, we recommend caregiver education on the signs of adrenal insufficiency. Greater vigilance may be indicated in those patients receiving the both types of hormone therapy in tandem. A routine post-hormone endocrinologist-guided laboratory assessment of adrenal function may not be feasible in all patients. We recommend prophylactic hydrocortisone when there is any concern for adrenal insufficiency, especially in times of stress. Further studies are needed to confirm the timing of a full recovery of the HPA axis in patients with infantile spasms treated with hormone therapy.

## Author Contributions

Both JM and SB made substantial contributions to the conception and design of this work as well as acquisition, analysis, and interpretation of the data, drafted the manuscript, made final approval of the version to be published, and are accountable for all aspects of this work.

## Conflict of Interest Statement

The authors declare that the research was conducted in the absence of any commercial or financial relationships that could be construed as a potential conflict of interest.

## References

[1] Lombroso CT A prospective study of infantile spasms: clinical and therapeutic correlations. Epilepsia (1983) 24(2):135–58. 6299719 10.1111/j.1528-1157.1983.tb04874.x

[2] Darke K Edwards SW Hancock E Johnson AL Kennedy CR Lux AL Developmental and epilepsy outcomes at age 4 years in the UKISS trial comparing hormonal treatments to vigabatrin for infantile spasms: a multi-centre randomised trial. Arch Dis Child (2010) 95(5):382–6. 10.1136/adc.2009.160606 20457702

[3] Mytinger JR Joshi S Pediatric Epilepsy Research Consortium, Section on Infantile Spasms. The current evaluation and treatment of infantile spasms among members of the child neurology society. J Child Neurol (2012) 27(10):1289–94. 10.1177/0883073812455692 22914371

[4] Hrachovy RA Frost JD Jr Kellaway P Zion TE. Double-blind study of ACTH vs prednisone therapy in infantile spasms. J Pediatr (1983) 103(4):641–5. 6312008 10.1016/s0022-3476(83)80606-4

[5] Baram TZ Mitchell WG Tournay A Snead OC Hanson RA Horton EJ. High-dose corticotropin (ACTH) versus prednisone for infantile spasms: a prospective, randomized, blinded study. Pediatrics (1996) 97(3):375–9. 8604274 PMC3100715

[6] Kossoff EH Hartman AL Rubenstein JE Vining EP. High-dose oral prednisolone for infantile spasms: an effective and less expensive alternative to ACTH. Epilepsy Behav (2009) 14(4):674–6. 10.1016/j.yebeh.2009.01.023 19435579

[7] Mytinger JR Weber A Heyer GL. The response to ACTH is determined early in the treatment of infantile spasms. Epileptic Disord (2015) 17(1):52–7. 10.1684/epd.2014.0723 25644547

[8] Lux AL Edwards SW Hancock E Johnson AL Kennedy CR Newton RW The United Kingdom infantile spasms study comparing vigabatrin with prednisolone or tetracosactide at 14 days: a multicentre, randomised controlled trial. Lancet (2004) 364(9447):1773–8. 15541450 10.1016/S0140-6736(04)17400-X

[9] Krasner AS Glucocorticoid-induced adrenal insufficiency. JAMA (1999) 282(7):671–6. 10517721 10.1001/jama.282.7.671

[10] Chamberlin P Meyer WJ III. Management of pituitary-adrenal suppression secondary to corticosteroid therapy. Pediatrics (1981) 67(2):245–51. 7243450

[11] Axelrod L Glucocorticoid therapy. Medicine (Baltimore) (1976) 55(1):39–65. 173974 10.1097/00005792-197601000-00003

[12] Plager JE Cushman P Jr Suppression of the pituitary-ACTH response in man by administration of ACTH or cortisol. J Clin Endocrinol Metab (1962) 22:147–54. 14487062 10.1210/jcem-22-2-147

[13] Perheentupa J Riikonen R Dunkel L Simell O. Adrenocortical hyporesponsiveness after treatment with ACTH of infantile spasms. Arch Dis Child (1986) 61(8):750–3. 3017239 10.1136/adc.61.8.750 PMC1777931

[14] Ross DL. Suppressed pituitary ACTH response after ACTH treatment of infantile spasms. J Child Neurol (1986) 1(1):34–7. 3036933 10.1177/088307388600100105

[15] Rao JK Willis J. Hypothalamo-pituitary-adrenal function in infantile spasms: effects of ACTH therapy. J Child Neurol (1987) 2(3):220–3. 3038989 10.1177/088307388700200309

[16] Riikonen R Donner M ACTH therapy in infantile spasms: side effects. Arch Dis Child (1980) 55(9):664–72. 6254450 10.1136/adc.55.9.664 PMC1627020

[17] Partikian A Mitchell WG. Major adverse events associated with treatment of infantile spasms. J Child Neurol (2007) 22(12):1360–6. 10.1177/0883073807310988 18174552

[18] Mytinger JR Quigg M Taft WC Buck ML Rust RS. Outcomes in treatment of infantile spasms with pulse methylprednisolone. J Child Neurol (2010) 25(8):948–53. 10.1177/0883073809356107 20142465

[19] Binder G Bosk A Gass M Ranke MB Heidemann PH. Insulin tolerance test causes hypokalaemia and can provoke cardiac arrhythmias. Horm Res (2004) 62(2):84–7. 10.1159/000079539 15249739

[20] Shulman DI Palmert MR Kemp SF Lawson Wilkins Drug and Therapeutics Committee. Adrenal insufficiency: still a cause of morbidity and death in childhood. Pediatrics (2007) 119(2):e484–94. 10.1542/peds.2006-1612 17242136

[21] Felner EI Thompson MT Ratliff AF White PC Dickson BA. Time course of recovery of adrenal function in children treated for leukemia. J Pediatr (2000) 137(1):21–4. 10.1067/mpd.2000.107385 10891816

[22] Mytinger JR Bowden SA Adrenal function testing following hormone therapy for infantile spasms: case series and review of literature. Front. Neurol (2015) 6:259 10.3389/fneur.2015.00259 PMC4672028 26696958

[23] Graber AL Ney RL Nicholson WE Island DP Liddle GW Natural history of pituitary-adrenal recovery following long-term suppression with corticosteroids. J Clin Endocrinol Metab (1965) 25:11–6. 14252277 10.1210/jcem-25-1-11

[24] Zöllner EW. Hypothalamic-pituitary-adrenal axis suppression in asthmatic children on inhaled corticosteroids: part 1. Which test should be used? Pediatr Allergy Immunol (2007) 18(5):401–9. 10.1111/j.1399-3038.2007.00540.x 17561933

[25] Rao RH Spathis GS. Intramuscular glucagon as a provocative stimulus for the assessment of pituitary function: growth hormone and cortisol responses. Metabolism (1987) 36(7):658–63. 3600280 10.1016/0026-0495(87)90150-8

